# Pancreatic Cancer and Cachexia—Metabolic Mechanisms and Novel Insights

**DOI:** 10.3390/nu12061543

**Published:** 2020-05-26

**Authors:** Kalliopi Anna Poulia, Panagiotis Sarantis, Dimitra Antoniadou, Evangelos Koustas, Adriana Papadimitropoulou, Athanasios G. Papavassiliou, Michalis V. Karamouzis

**Affiliations:** 1 Department of Nutrition and Dietetics, Laiko General Hospital, 11527 Athens, Greece; lpoulia@gmail.com; 2 Molecular Oncology Unit, Department of Biological Chemistry, Medical School, National and Kapodistrian University of Athens, 11527 Athens, Greece; panayotissarantis@gmail.com (P.S.); vang.koustas@gmail.com (E.K.); papavas@med.uoa.gr (A.G.P.); 3 Oncology Department of Daily Hospitality, Laiko General Hospital, 11527 Athens, Greece; demiantoniadou@gmail.com; 4 Center of Basic Research, Biomedical Research Foundation of the Academy of Athens, 11527 Athens, Greece; adapapadim@gmail.com; 5 First Department of Internal Medicine, Laiko General Hospital, Medical School, National and Kapodistrian University of Athens, 11527 Athens, Greece

**Keywords:** pancreatic cancer, cachexia, systemic inflammatory response

## Abstract

Cachexia is a major characteristic of multiple non-malignant diseases, advanced and metastatic cancers and it is highly prevalent in pancreatic cancer, affecting almost 70%–80% of the patients. Cancer cachexia is a multifactorial condition accompanied by compromised appetite and changes in body composition, i.e., loss of fat. It is associated with lower effectiveness of treatment, compromised quality of life, and higher mortality. Understanding the complex pathways underlying the pathophysiology of cancer cachexia, new therapeutic targets will be unraveled. The interplay between tumor and host factors, such as cytokines, holds a central role in cachexia pathophysiology. Cytokines are possibly responsible for anorexia, hypermetabolism, muscle proteolysis, and apoptosis. In particular, cachexia in pancreatic cancer might be the result of the surgical removal of pancreas parts. In recent years, many studies have been carried out to identify an effective treatment algorithm for cachexia. Choosing the most appropriate treatment, the clinical effect and the risk of adverse effects should be taken under consideration. The purpose of this review is to highlight the pathophysiological mechanisms as well as the current ways of cachexia treatment in the pharmaceutical and the nutrition field.

## 1. Introduction

Cachexia is a multifactorial syndrome characterized by non-volitional weight loss, sarcopenia and adipopenia, fatigue, weakness, loss of appetite, and early satiety. The term derives its origin from the Greek terms “κακόςkakos” and ἕξιςhexis,” meaning “bad” and “condition,” respectively. Cachexia occurs in multiple non-malignant diseases, i.e., Human Immunodeficiency Virus (HIV)/Acquired Immunodeficiency syndrome (AIDS), rheumatoid arthritis, cardiac failure, chronic kidney disease, and cancer; the latter of which will be the focus of the present review [1]. Cancer cachexia, i.e., the cachexia observed in cancer patients, is multifactorial and encompasses both physiological and psychological etiologic factors. It affects approximately 50% of all cancer patients and is driven by reduced food intake, alongside with specific alterations in the complex hormonal network regulating metabolism, inducing elevated energy expenditure, excess catabolism, and inflammation. Therefore, cachexia differs significantly from starvation resulting from energy deprivation, as it is not easily reversible with the provision of nutrients as the pathophysiological background should also be taken into account [2,3].

Cachexia is associated with a worse prognosis and therefore it affects negatively overall survival. Approximately 20% of all cancer deaths can be attributed to Cachexia. It significantly deteriorates the patients’ quality of life (QoL) and at the same time, it aggravates chemotherapy side effects. Cancer affecting upper gastrointestinal system (GI) and pancreas have the highest rates of cancer cachexia, with almost 80% of those in terminal state manifesting it [3]. In addition, cachexia is closely correlated to 33% of PC deaths and, in combination with anemia and/or chronic inflammation, may lead to fatigue, an immunosuppressive tumor environment, and inhibition of chemotherapy tolerance [4].

The diagnostic criteria for cancer cachexia include on the percentage of weight loss in a certain timescale, in combination with the presence of a Body Mass Index (BMI) below the normal cutoffs. Cachexia is defined by involuntary weight loss greater than 5% of the usual body weight or weight loss of more than 2% in those with a BMI at baseline minor than 20 kg/m^2^, over six months [5]. Moreover, the occurrence of sarcopenia (skeletal muscle malfunction and/or depletion) accompanied with any grade of weight loss greater than 2% of the usual body weight should be considered as cachexia. Sarcopenia, according to the most recent diagnostic criteria, can be detected through dynamopenia (criterion 1) and then diagnosed by the low muscle mass (criterion 2) and low physical performance status (criterion 3) [6]. The evaluation of muscle mass quality and quantity can be performed by: anthropometric measurements, i.e., by the measurement of mid-upper-arm muscle area (with a cutoff of < 32 cm^2^ for men and < 18 cm^2^ for women), by dual-energy X-ray absorptiometry and the evaluation of appendicular skeletal muscle index (cutoff: men < 7.26 kg/m^2^, women < 5.45 kg/m^2^), by oncology computed tomography (CT) imaging and the estimation of lumbar skeletal-muscle index (cutoff for men < 55 cm^2^/m^2^ and for women < 39 cm^2^/m^2^), and by bioelectrical impedance by which whole-body fat-free mass index without bone can be determined (men < 14.6 kg/m^2^, women < 11.4 kg/m^2^) [7].

Cancer cachexia comprises three sequential clinical stages: pre-cachexia, cachexia, and refractory cachexia. At the pre cachectic stage, patients experience metabolic alterations such as loss of appetite and impaired glucose metabolism before any significant unintentional weight loss. Patients who experience continuous significant weight loss according to the aforementioned criteria are candidates for developing cachexia. Cachexia is considered as clinically refractory when cancer is pre-terminal or when the patient is not responding to anticancer therapy. For patients at that stage, with a life expectancy not exceeding the three months and therapeutic interventions are most of the times palliative on [8,9].

Cachexia is a frequent and prominent feature of pancreatic cancer (PC), being present even by the time of diagnosis, as 85% of PC patients experience a reduction in their body weight, whereas close to the terminal phase, the median weight loss can reach 25% of the pre-illness weight [10]. As the tumor’ metabolism is highly energy- and nutrient-consuming, higher dietary intakes are required in order to sustain energy and metabolic intermediates that are necessary for the sustainability of proliferation and the overall survival of the “host” of the PC cancer. It is not surprising, therefore, that the changes induced by the tumor can affect the patients’ body not only on a cellular level but also its overall survival.

## 2. Mechanisms and Pathophysiology of Cancer Cachexia

**Figure 1:**
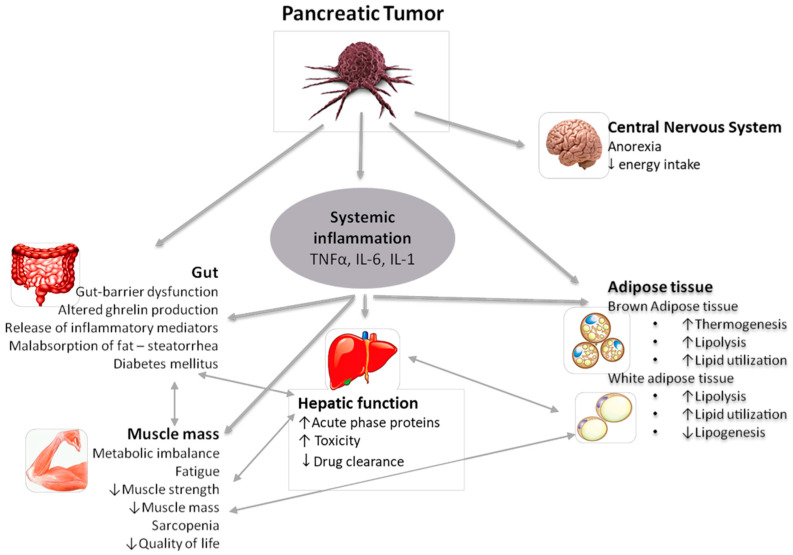
Pathophysiology of Pancreatic Cachexia: Organs, molecules, and metabolic dysregulations. TNF-α: tumor necrosis factor α; IL: interleukin.

Cancer has the ability to alter the homeostatic mechanisms of energy balance in the human body. Cancer cachexia is a complex multifactorial syndrome involving tumor and host-derived signaling factors and abnormal metabolism that finally leads to muscle mass deterioration [4]. Cancer cachexia includes the participation of various different molecules such as cytokines, hormones, neuropeptides/neurotransmitters, and tumor-derived factors [11,12] (Figure 1).

### 2.1. Mechanical Factors

Patients with PC are at high nutritional risk due to factors other than cancer-associated cachexia. The anatomic alteration due to the tumor, i.e., the extrinsic compression from the tumor causing mechanical obstruction of the GI tract, can directly cause pain or induce symptoms that affect nutritional intake or absorption i.e., fatigue, dysphagia, gastroparesis, pancreatic insufficiency, malabsorption, and constipation. Moreover, the cancer-induced pancreatic insufficiency, leading to exocrine (steatorrhea) and endocrine (diabetes mellitus or DM) disturbances, can disrupt the energy balance either by increasing nutrient losses (mainly fatty acids) in in defecation or by imposed restrictions for the DM management and control. Finally, the surgical resection of the tumor on the pancreatic head, i.e., a pancreatic duodenectomy, may exacerbate pancreatic insufficiency and reduce oral intake [13,14]. As a result, PC patients usually experience a significant loss of body weight by the time of diagnosis and usually are the ones that it is more likely to have the worst prognosis [15].

### 2.2. Inflammation

As mentioned before, cachexia is different from simple starvation. Starvation causes fat tissue depletion, while the weight loss in a cachectic patient is attributed both to muscle and fat mass loss. This is attributed to hyper catabolism of muscle mass that leads to a negative balance protein mass. Moreover, this is enforced by the imbalance in protein metabolic pathways, which is one of the commonest manifestations of cancer cachexia [16].

The hyper catabolism of cancer cachexia is ascribed mainly to systemic inflammatory response caused by the tumor itself, which promotes fat and protein catabolism. Upregulated C-reactive protein (CRP) levels (CRP > 10 mg/L) have been observed in cachectic patients with cancer and it is connected with poor performance in PC patients. In the same context, elevated levels of the cytokines interleukin-6 (IL-6) and interleukin-10 (IL-10) are correlated with weight loss and worse clinical outcomes. Tumorigenesis leads to cytokines release, either by the tumor or the host and eventually to metabolic pathways resulting in anorexia and hyper-catabolism [17].

A subclinical inflammation with increased C-reactive protein (CRP) is observed in many patients with solid tumors, including PC. It contributes to elevated energy expenditure and anorexia. In PC-dependent cachexia, interleukin 1 (IL-1), interleukin 6 (IL-6) Interleukin 8 (IL-8), and tumor necrosis factor α (TNF-α) are the most common pro-inflammatory cytokines [18]. PC tumor cells are the primary source of cytokines such as IL-6, which is significantly upregulated by peripheral blood mononuclear cells (14-fold) and is associated with PC patients’ reduced survival [19].

Cytokines possibly act as an inhibitor for the neuropeptide Y pathway or mimic the adverse feedback action of leptin on the hypothalamus, causing a loss of appetite. Pro-inflammatory cytokines such as TNF-α and IL-1 act directly in the central nervous system (CNS), resulting in an anorexigenic effect. It has been proposed that these two molecules have a synergistic role, as TNF-α upregulates IL-1 secretion and both stimulate many cytokines, such as IL-6, in a manner of cascade [20,21]. Moreover, the pro-cachectic factor TNF-α has been linked with malnutrition in patients with PC. TNF-α has been shown to boost lipolysis, downregulate lipogenesis, and stimulate the catabolism of lean body mass. IL-6 and TNF-α stimulate signaling pathways that eventually result in skeletal muscle protein degradation, the two most characterized being the Janus kinase (JAK)-signal transducer and activator of transcription (STAT) or Jak2/STAT3 pathway and the Nuclear Factor kappa-light-chain-enhancer of activated B cells (NF-Κb) pathway [22].

The JAK-STAT signaling pathway is activated by a variety of cytokines, involved in signal transduction and the mediation of inflammation, cancer progression, muscle mass wasting, weight loss, and cancer cachexia [23]. NF-κB activation, as an oxidative stress-dependent product, upregulates the degradation of ubiquitin-mediated proteasome [24]. Chemotherapy stimulates NF-κB activation, a factor that regulates lean body mass wasting. Therefore, one of the effects of chemotherapy on PC patients would the activation of NF-κB, leading to wasting [25,26]. Activation of NF-κB might upregulate transcriptors that suppress the transcription of myoblast determination protein 1 (MyoD), a protein connected to the satellite cell proliferation after muscle injury, resulting in a reduction of the ability of muscle cells to repair themselves. [27]. Although skeletal muscle loss is a prerequisite for the clinical diagnosis of cancer cachexia, lipolysis and adipopenia may occur to an extent before muscle loss inducing a vicious circle: lipolysis results in increased free fatty acids in circulation, ending up in skeletal muscle, where they trigger the secretion of ubiquitin ligases Atrogin-1 and MuRF1, and consequently, the skeletal muscles atrophy [28]. During this, a sequence of events occurs including activation of proteolytic systems and impeding of the activity of contractile proteins and organs, leading in the shrinkage of muscle fibers. Recent studies have demonstrated un unexpected correlation between TNFα and myogenin on MuRF1 and atrogin-1 expression.More specifically, TNFα treatment leads to elevated levels of myogenin, MuRF1 and atrogin-1, by blocking the TNFα-mediated myogenin upregulation [29] while inducing the production of muscle-specific ubiquitin ligases. However, the exact mechanisms of TNFα-mediated myogenin regulation and the its involvement in muscular deficits are not yet fully elucidated [30].

### 2.3. The Role of Adipose Tissue

Enhanced thermogenesis from brown adipose tissue (BAT) also seems to have an essential role in cancer cachexia. In adult patients, BAT adipocytes contain multilocular lipid droplets that produce energy through the initiation of a proton leakage pathway in the inner mitochondrial membrane, which is regulated by uncoupling protein (UCP) 1 [31].

UCPs regulate mitochondrial proton gradients and take part in the production of reactive oxygen species in skeletal muscle. UCP1 is localized only in BAT in contrast with UCP2, which is found almost in all tissues [32]. UCP3 is detected in both BAT and skeletal muscle. UCP2 and 3 have been identified to correlate with metabolic activity and energy use in skeletal muscle [33]. Moreover, experimental evidence correlates high levels of both proteins with the cachectic state for cancer patients. In mice models, increasing UCP1 mRNA levels are identified in BAT over controls with cancer cachexia. Besides that, UCP2 and -3 levels appear to increase in skeletal muscle but not in BAT [31].

Lipolysis plays a substantial role in the pathogenesis of cancer cachexia. The increased catabolism of the stored fat leads to a complete loss of white adipose tissue (WAT) followed by a reduction of muscle mass. The lack of adipose triglyceride lipase (Atgl) and—to a lesser extent—hormone sensitive lipase (Hsl) reduces the decomposition of fatty acids (FAs) and maintains WAT and muscle mass, preventing cachexia [34].

### 2.4. Tumor-Derived Factors

Tumor-derived factors are also associated with the regulation of the metabolic abnormalities, which lead to PC cachexia. Two of the most well-known molecules secreted from the cancer cells are the lipid mobilizing factor (LMF) and the proteolysis-inducing factor (PIF).

### 2.5. Lipid Metabolism

LMF production is elevated in cachexia-inducing tumors in combination with the increased oxidation of fatty acids via UCP stimulation, results in the degradation of the adipose tissue [35]. Taking into account that LMF was found only in cancer patients bearing weight loss and not in others with normal weight, it was considered to be a serum protein that could act as potential marker for PC cachexia. It has been shown to promote GTP-dependent lipolysis mediated by β3 adrenergic receptors while enhancing the response of adipose tissues to the lipolytic effects of other stimuli such as catecholamines [36].

Moreover, Surface-Enhanced Laser Desorption (SELDI) was used in order to analyze the serum from PC patients with cachexia. In PC-dependent cachectic patients, the levels of glucagon-like peptide-1 (GLP-1), apolipoprotein C-II and III, were increased. GLP-1 appeared to trigger satiety and inhibit food intake through molecular modulation on the hypothalamus [37]. Both lipoproteins regulate lipid metabolism and are closely correlated with lipogenesis inhibition and negative energy balance. Moreover, increasing levels of zinc-α2-glycoprotein (ZAG), a stimulator of lipolysis, have also been identified in the current study [38,39]. Therefore, dysregulation of lipid metabolism plays a crucial role in the pathogenesis of PC cachexia.

### 2.6. Proteolysis-Inducing Factor (PIF)

PIF, originally isolated from a murine tumor, was found to stimulate skeletal muscle catabolism in experimental models. PIF is present in cachectic cancer patients and the urine of 80% of PC patients with high total weight loss. PIF is thought to promote a decrease in muscle mass, increasing protein degradation and a decrease in protein synthesis in gastrocnemius muscle [40]. The activation of the ubiquitin-proteasome pathway (UPP) and NF-κB in skeletal muscle is possible to contribute to protein degradation [41]. PIF activates double-stranded RNA-dependent protein kinase (PKR), a process that eventually inhibits translation and, subsequently, protein synthesis [42].

Τhe ATP-dependent UPP is considered to be the most important for the degradation of myofibrillar proteins in cancer cachexia. The normal function of UPP is to remove excess or damaged proteins in mammalian cells, regardless of the dietary supply of protein. UPP may be activated in cachectic patients by the tumor-derived substance PIF. An experimental study showed that intravenous administration of PIF to healthy mice resulted in upregulated mRNA levels for ubiquitin, ubiquitin transporter protein E2, and the C9 proteasome subunit, as well as a rapid reduction in body weight [43].

### 2.7. The Role of the Hypothalamus and Other Central Pathways

The management of appetite and efficient food consumption is vital in cancer patients suffering from cachexia, as it supports the maintenance of body weight and consequently improves a patient’s life quality. A primary mechanism in cancer-depended cachexia occurs through dysregulation of the hypothalamic axes related to energy uptake such as neuropeptide Y (NPY) and proopiomelanocortin (POMC)/cocaine pathways. Specific hormones are responsible for either inhibiting food intake (insulin leptin, peptide YY/GLP1, and cholecystokinin) or increasing it (ghrelin).

Ghrelin is an orexigenic peptide with significant appetite-inducing effect and is also responsible for Growth Hormone stimulation and gastric motility [44]. Hence, ghrelin could be used as a novel therapeutic strategy to manage cancer cachexia, as it has been demonstrated to improve symptoms in tumor-bearing animal models and patients with cancer [45,46].

In addition, cancer cachexia may be triggered through IL-1 and other pro-inflammatory cytokines that activate the POMC/CART pathways, leading to a significant anorexic effect.

Low leptin levels due to body fat depletion stimulate energy intake via the NPY/AgRP pathway. Nevertheless, TNF-α and IL-1 upregulate leptin mRNA expression in adipocytes and plasma of cancer patients despite decreased adiposity, disorganizing the physiological compensatory mechanisms [47].

Serotonin may also play a pivotal role in the development of cancer anorexia through the melanocortin system. In experimental models, IL-1 induces the release of hypothalamic serotonin. Upregulated serotonin levels, in turn, contribute to the persistent activation of POMC/CART neurons, resulting in decreased appetite and anorexia [48].

### 2.8. Insulin Metabolism and Insulin Resistance

In cancer cachexia, elevated endogenous glucose production with up-regulated gluconeogenesis and insulin resistance has been reported, but different with Type 2 Diabetes (T2D), fasting glucose most of the times within normal values. The chronic inflammatory state of cachectic patients may result in pancreatic b-cell dysfunction and in combination with the negative effect of PC on pancreas functionality an impaired insulin secretion may be observed [49]. Moreover, malignant cells of active neoplasms rely mainly on glycolysis for the production of energy, a metabolic path that is 18 times less efficient than oxidative phosphorylation in regards to ATP production. The production of energy by glycolysis results to the pyruvate and then lactate, even in aerobic conditions, which then is recycled to glucose by hepatic or other tissue metabolism, by an inefficient Cori Cycle This metabolic alteration is known as Wartburg effect [50,51].

Insulin resistance in known to negatively affect protein anabolism in elderly and T2D patients [52]. The negative effect of insulin resistance in muscle mass in cancer patients has been shown to exist in animal studies, where in a mouse model with colon adenocarcinoma, insulin resistance was profound even before the muscle or weight loss [53]. In humans, in a study of non-small cell lung cancer (NSCLC) patients with moderate weight loss, protein anabolism was negatively affected by insulin resistance, an observation that was correlated with their inflammatory status but not with the weight loss itself [54]. Adding to that, insulin resistance and its interaction with ATP-dependent UPP via caspase-3 have been identified as another possible mechanism contributing to protein degradation [55].

Finally, experiments in Drosophila melanogaster show that intestinal activation of Yorkie, leads to increased proliferation and the secretion of ImpL2 an insulin growth factor binding protein (IGFBP) that inhibits both insulin and insulin-like growth factor-1 (IGF-1) signaling [56]. Additionally, ImpL2is produced directly by different tumor types, developing peripheral organs insulin resistance [55,57]. On the other hand, the insulin/IGF-1 signaling seems to be elevated in cancer cells, consequently allowing the advantage of systemic hyperglycemia [58].

### 2.9. Neural Invasion

PC neural invasion may result in nerve damage from intraneural tumors on site. The subsequent astrocyte activation in the spinal cord further stimulates the sympathetic nervous system, which in turn induce lipolysis and muscle atrophy [59].

### 2.10. Zinc Deficiency

Zinc is a trace metal that constitutes a vital component of many enzymatic complexes and transcription factors. Despite the requirement of zinc in many significant cellular processes, zinc uptake and storage are strictly regulated as the increased levels could be detrimental for the cell. It has been shown that tumor cells exhibit dysregulated zinc uptake and efflux, and in some malignancies, this abnormal transport is an index of tumor viability [56]. Cancer disrupts zinc metabolism as the consequence of the acute phase reply to the inflammatory cytokine activity. Numerous mechanisms are resulting in zinc deficiency in PC patients: low albumin decreasing zinc-binding capacity, reduced nutrient intake due to anorexia, progressive loss in muscle and gastrointestinal cells from ubiquitin-proteasome activation, and augmented urinary excretion of zinc. Moreover, as zinc deficiency causes hypogeusia, it contributes to the low appetite for food and anorexia in cancer patients [60]. In addition, there is a crucial role for the metal ion transporter, ZIP14, in cachexia related to colon, breast, lung, and PC. ZIP14 increases the levels of intracellular zinc in muscle cells hence disturbing and dearranging zinc homeostasis [61].

## 3. Current Treatment Options

The clinical management of cachexia in PC has certain limitations and is characterized by extreme complexity. First of all, it is vital to control the factors and symptoms of the disease and its clinical symptoms that induce anorexia, such as pain, nausea, pancreatic insufficiency, and constipation. For the prevention and the management of cancer cachexia, pharmacotherapy and nutritional support are combined in order to control the side effects of the treatment, the symptoms of the disease and provide the macronutrients the patient needs to maintain his nutritional status.

### 3.1. Pharmacological Treatment

#### 3.1.1. Progesterone, Corticosteroids, Anti-Inflammatory Drugs

The role of progesterone is to stimulate the appetite via direct and indirect pathways in the CNS. Progestogens, such as megestrol acetate (MA) and corticosteroids, are used as orexigenic agents [62]. Their mechanism of action is through the inhibition of cytokines and restoration of appetite, resulting in weight gain, as seen in recent animal and human models [63], but not always accompanied by respective improvements in quality of life in humans [64].

Corticosteroids reduce prostaglandin activity and suppress pro-inflammatory cytokines such as IL-1 and TNF-α. At the same time, they have a positive effect on mood and appetite, but these effects are usually short term, not lasting more than a month. It should also be in mind that long term provision of corticosteroids are linked with considerable adverse effects, such as osteoporosis, myopathy, glucose metabolism imbalance and an increased risk of infections [65].

Several studies on nonsteroidal anti-inflammatory drugs (NSAIDs), such as indomethacin and ibuprofen, highlight the positive effect on patients’ cancer-dependent cachexia. Furthermore, studies on NSAID agents, omega-3 fatty acids, and thalidomide highlight the inhibition of inflammatory response through the alteration of cytokine production. The combination therapy, which encompasses NSAID, dietary consultation, nutritional supplementation, and exercise, has already shown beneficial results for lung or PC patients [66].

Multiple studies have demonstrated that inhibition of the JAK2-STAT3 signaling pathway can alleviate cancer cachexia and skeletal muscle wasting by inhibiting the inflammatory response [23,67]. Moreover, the Jak2/Stat3-dependent signaling pathway plays a key role since its pharmacological inhibition with AG490 (JAK/STAT3 pathway) strongly attenuates cachexia progression in a lethal transgenic PC mice model. Ruxolitinib, another JAK2 inhibitor, combined with capecitabine may improve survival in patients with metastatic PC and profound systemic inflammation [68].

#### 3.1.2. Anti-Cytokine Treatment

TNF-α promotes lipolysis and myopenia. The drug thalidomide, which reduces the production of TNF-α and other pro-inflammatory cytokines, was shown to be efficient in the management of cancer cachexia in patients with gastrointestinal and PC [69]. Nevertheless, thalidomide has substantial adverse effects, which should be closely monitored and require careful risk-benefit analysis. Lenalidomide, a derivate of thalidomide, is an immunomodulatory drug and its primary effect is the decrease of inflammatory cytokines. As a cancer cachexia syndrome treatment, thalidomide has been shown to stabilize lean body mass in a randomized controlled trial [70]. Anti-TNF-α antibodies, on the other hand, such as infliximab and etanercept, did not exhibit any significant improvements in cachectic patients and its use was accompanied by many side effects [71].

IL-6 alters the cachectic patient recovery by targeting several tissues, such as skeletal muscle, liver tissue, gut, and adipose. Increased levels of IL-6 are correlated with sarcopenia, severe fatigue, and accelerated weight loss [72]. Monoclonal antibodies (MoAbs) against IL-6, such as clazakizumab, have been identified as a putative treatment for cachexia in patients with NSCLC [13,23]. Several clinical studies treat cancer patients with tocilizumab, a MoAb against humanized IL-6R. Tocilizumab appears to reduce plasma levels of IL-6, attenuate muscle loss, and restore the plasma albumin levels without affecting the proliferative tumor rate [73]. Finally, leukemia inhibitory factor (LIF) secreted from the tumor contributes to cancer cachexia by increasing the levels of IL-6, indicating that blockage of LIF with an antibody or by gene silencing, can relief the symptoms of cachexia [34].

Clinical trials (ClinicalTrials.gov numbers NCT01505530 and NCT01433263) evaluated the effect of molecules that target cytokine activity and myostatin (muscle growth inhibitor) but without a report of a significant benefit up to date. Thus, newer therapies (NCT03207724) with monoclonal antibodies (MoAbs) against essential inflammatory cytokines, such as IL-1 alpha, have already been recruited in a phase II/III clinical study (MENAC, NCT02330926) [74].

In the last years, several studies have identified the effect of transforming growth factor-beta (TGF-β1) cachexia and anorexia in mice models [75]. Limited clinical data highlight the potential role of the use of trabedersen (a TGF-β2 antagonist) in increasing the overall survival in PC patients. This mechanism probably involves the disruption of tumor cytokine secretion and the upregulation of host antitumor cytokines [76].

#### 3.1.3. Ghrelin

Ghrelin has many activities such as inhibition of apoptosis, regulation of differentiation, and stimulation or inhibition of proliferation of several cell types [77]. Novel agents for appetite stimulation include ghrelin and ghrelin mimetics, namely anamorelin, which use resulted in a significant increase in lean body mass in non-small cell lung cancer patients from two trials in phase III [78]. Ghrelin transmits hunger signals from the periphery to the CNS, resulting in elevated appetite. Moreover, they induces growth hormone release, blocks muscle catabolism and stimulates gut motility, thus contributing to weight maintenance and/or increase [34,79]. Acylated and unacylated ghrelin induce the direct activation of antiatrophic pathways at the skeletal muscle, resulting to a reduction of muscle loss [80].

Adverse effects related to anamorelin were reported and they were mainly hyperglycemia, nausea, and dizziness. Existing evidence, though, suggests that short-term administration of synthetic ghrelin appears to be safe and well-tolerated [81,82].

#### 3.1.4. Hormones

It is known that throughout cachexia, signaling of insulin, IGF-1, and Growth hormone (GH) is dysregulated. The role of insulin signaling in preventing cachexia is further demonstrated by experiments showing that mice treated with insulin sensitizers (rosiglitazone) and patients treated with insulin ameliorate cachexia symptoms [81]. GH promotes the production of IGF-1 in the liver and other tissues typically. IGF-1 interacts with insulin to modulate the control of carbohydrate metabolism [82]. Low serum concentrations of IGF-1 are present in cachectic patients, while there seems to be a peripheral GH and Insulin resistance, which leads to a negative amino acid balance in skeletal muscle. The therapeutic use, though, of Insulin, GH, or IGF-1 is currently not recommended due to massive side effects [83]. GH/IGF-1 provides an anti-apopototic environment that could eventually accelerate the development of cancer [84,85].

Testosterone and its synthetic derivates are anabolic steroid hormones. They have a positive effect on muscle mass through the upregulation of protein synthesis. Low doses of testosterone increase insulin sensitivity, but on the other hand, high doses increase insulin resistance. Testosterone may be considered as a possible therapeutic option for cancer cachexia. In order to study this, α double-blind study is currently in progress (NCT00878995), where the side effects reported are liver defects and cardiovascular disorders [84]. Another hopeful approach is a treatment with selective androgen receptor modulators (SARMs). SARMs correspond with androgen-receptors in the muscle tissue only, minimizing the systemic side effects of androgen treatment. Ostarine has been tested with promising results in Phase I and II clinical trials and it has been shown that they may have acted as a potent anabolic agent with minimal side effects [85]. Similarly, Enobosarm, a SARM under investigation, has already been used to treat other muscle-wasting diseases. In a Phase III clinical study, enobosarm was found to improve muscle function and increase lean body mass in patients with NSCLC. However, another clinical study NSCLC did not confirm the effects of enobosarm on muscle loss of patients with cancer cachexia [86].

#### 3.1.5. Cannabinoids

Cannabinoids boost appetite and food intake in PC patients. At the same time, the synthetic delta-9-tetrahydrocannabinol (D-9 THC), dronabinol, has been permitted for use in AIDS-related cachexia and emesis from chemotherapy. In a pilot study, it has been shown that, patients treated with THC reported increased premeal appetite, quality of sleep, caloric intake, relaxation, and improvements in their taste compared to the patients under placebo therapy [87]. Despite these findings, the available data for the use and effectiveness of cannabinoids for the treatment of cancer cachexia is insufficient, and therefore, their systematic use in clinical practice cannot yet be supported [88].

#### 3.1.6. Pancreatic Enzyme Replacement Therapy (PERT)

Pancreatic exocrine deficiency is one of the main factors contributing to PC related malnutrition. Pancreatic exocrine insufficiency occurs mainly in resectable cancers after surgery and in advanced PC patients. PERT has been shown to induce weight gain, limit weight loss [89], reduce diarrhea and steatorrhea, and improve pancreatic pain and bloating/gas symptoms [90]. Finally, some data support their positive effect on survival in patients with unresectable tumor [91].

### 3.2. Nutritional and Lifestyle Management for Patients with Pancreatic Cancer Cachexia

Cancer-induced cachexia constitutes one of the main factors of morbidity and mortality in cancer treatment; therefore, the establishment of efficient nutritional support modalities is of significant importance. According to the recent guidelines of clinical nutrition in oncology [92], the aim of nutrition interventions is the maintenance or the improvement of nutritional intake, the management of the metabolic alterations, the maintenance of muscle mass and functionality, and the reduction of the risk of toxicity that could cause cancelation of the anticancer treatments and deteriorate the patients quality of life (QoL).

In PC patients, where nutrition deficits are common, nutrition interventions should start early to prevent excessive deficits. The first line of nutritional support is nutrition counseling by a specialized dietitian/nutritionist or nutrition specialist who can provide a thorough nutritional assessment and nutrition advice aiming to help patients to maintain or increase energy and protein intake, preferably with regular food [93]. Sufficient energy and protein intake reaching 35–40 Kcal/Kg/day of non-protein energy and 0.25 to 0.3 g/Kg/day of nitrogen is considered to pose significant effects on morbidity and mortality [94]. Moreover, experiments in mice show that Ketogenic diet might be linked with reduced tumor growth, less metastatic spread, extended survival, and metabolic alterations that act prophylactively against cancer cachexia [95,96]. Prolepsis and management of steatorrhea by the provision of dietary advice on fat intake reduction and supplementation of fat soluble vitamins in case of malabsorption, in combination with pancreatic enzymes supplementation [97,98] are of vital importance, as fat loss in defecation has an adverse effect on the energy balance of the patients. Moreover, the nutritional management of DM can also be helpful for patients with disturbed blood glucose levels due to the tumor or the pancreatic resection. For patients with pancreatectomy, dedicated sessions on carbohydrate counting and insulin management are also crucial.

As anorexia and nausea are common symptoms in PC patients, nutritional advice alone oftentimes fail to provide significant results. The provision of oral nutritional supplements (ONS), i.e., commercially available nutritionally complete nutrient mixtures, is usually recommended to supplement voluntary nutritional intake [92]. ONS for PC patients should be chosen based on each patient’s individual needs and should always consider the management of the symptoms of the disease. Moreover, as systemic inflammation—commonly present in cancer patients—have a hypercatabolic effect, the current nutritional strategies now focus on nutritional parameters with anti-inflammatory action. The addition of the essential amino acid (EAA) of leucine may have a positive impact on protein metabolism, even when inflammation is present [97]. Fish oil, the primary source of n-3 fatty acids, is currently suggested as a way to downregulate inflammation, and therefore, it is considered a significant way to improve appetite, food intake, lean body mass, and body weight in patients with cancer cachexia. According to the results of a randomized control study in patients with advanced colorectal cancer, the provision of 2 g of fish oil daily during the first nine weeks of chemotherapy resulted in longer time-to-tumor progression [98]. While studies are still required to confirm the positive effects of n-3 fatty acids, fish oil remains a promising nutrient in nutrition management of cancer patients. Especially for patients with pancreatic insufficiency, n-3 fatty acids should be taken along with pancreatic enzymes in order to ensure sufficient absorption and better tolerance from the patient.

The main problem with ONS in cancer patients is compliance and the fact that—due to their satiating effect—they often limit significantly the proportion of the food consumed at mealtimes. If voluntary nutritional intake is insufficient to support patients’ nutritional status, provision of complete nutritional support, either enteral or parenteral, should be considered, depending on the level of GI functionality. Enteral nutrition refers to the provision of nutrients through enteral tubes (nasogastric or nasojejunal tube, gastrostomy, or jejunostomy). Parenteral nutrition, i.e., the provision of nutrients directly in the circulation, either through a central vein or a peripheral one, may be recommended in cases of complete bowel obstruction or intolerance of the enteral nutrition support [2,94].

**Figure 2:**
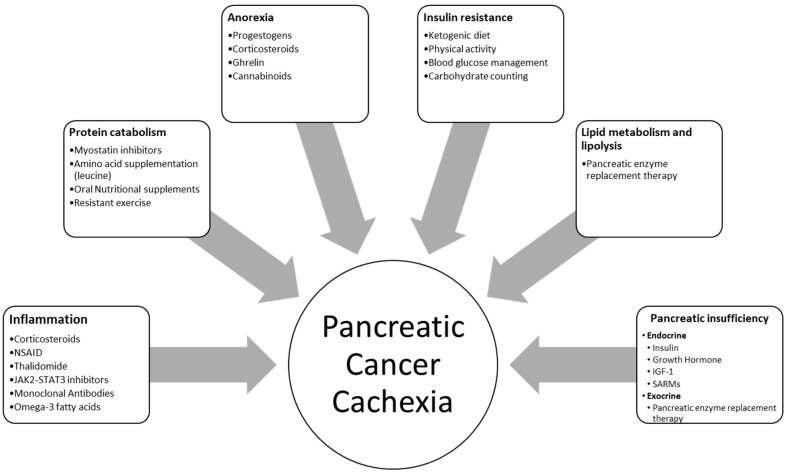
Conditions which are linked with pancreatic cancer cachexia and their therapeutic management. NSAID: nonsteroidal anti-inflammatory drugs; JAK: Janus kinase; STAT: signal transducer and activator of transcription; IGF: insulin-like growth factor; SARMs: selective androgen receptor modulators.

Another parameter that should be considered is physical activity. Cancer patients are prone to physical inactivity due to psychological reasons (i.e., depression) or physical restrains (i.e., fatigue, adverse effects of the anticancer treatments). Provision of sessions of physiotherapy, including activities of daily life, resistance, and aerobic exercise, should be prescribed to maintain and increase muscle mass and/or muscle functionality and strength. In that context, physiotherapy can promote anabolism and retain a utilization of nutrients, especially protein [92] (Figure 2).

## 4. Conclusions

Cancer cachexia is a multifactorial condition and is highly prevalent in patients with PC. The tumor-induced abnormalities in muscle metabolism and the physiological and functional imbalances of the pancreas are among the etiological factors for the muscle wasting associated with cachexia and the decrease of the overall prognosis of PC patients. For the effective management of patients with PC, a multimodal approach should be implemented, aiming to tumor growth inhibition through anticancer therapy and improve the overall life quality of the patient. The early detection of signs and treatment of symptoms that could have a negative impact on the nutritional status of the patient is of paramount importance in order to identify patients in the initial stages of the disease even at the stage of precachexia. Management of inflammation, hormonal therapy, PERT, and individualized nutritional care can be combined for the maximal possible effect. Further studies are required for the provision of solid data regarding the safety and the efficacy of the current or novel treatments of cancer cachexia in order to provide efficient therapies with the minimum possible risk for the patients.
